# Red blood cell lipid distribution in the pathophysiology and laboratory evaluation of chorea-acanthocytosis and McLeod syndrome patients

**DOI:** 10.3389/fphys.2025.1543812

**Published:** 2025-03-27

**Authors:** Anne-Sophie Cloos, Marine Ghodsi, Amaury Stommen, Steffen M. Recktenwald, Lars Kaestner, Adrian Danek, Adrian Spranger, Andreas Hermann, Kevin Peikert, Donatienne Tyteca

**Affiliations:** ^1^ CELL Unit and PICT imaging Platform, de Duve Institute, Brussels, Belgium; ^2^ Dynamics of Fluids, Experimental Physics, Saarland University, Saarbrücken, Germany; ^3^ Micro/Bio/Nanofluidics Unit, Okinawa Institute of Science and Technology Graduate University, Okinawa, Japan; ^4^ Theoretical Medicine and Biosciences, Medical Faculty, Saarland University, Homburg, Germany; ^5^ Neurologische Klinik, Ludwig-Maximilians-Universität, Munich, Germany; ^6^ Department of Neurology, Translational Neurodegeneration Section “Albrecht Kossel”, University Medical Center Rostock, University of Rostock, Rostock, Germany; ^7^ Center for Transdisciplinary Neurosciences Rostock (CTNR), University Medical Center Rostock, Rostock, Germany; ^8^ Deutsches Zentrum für Neurodegenerative Erkrankungen (DZNE) Rostock/Greifswald, Rostock, Germany; ^9^ United Neuroscience Campus Lund-Rostock (UNC), Rostock, Germany

**Keywords:** acanthocytes, lipid domains, cholesterol, ceramide, sphingomyelin, spectrin cytoskeleton, erythrocyte maturation, microfluidics

## Abstract

The core neuroacanthocytosis syndromes, *i.e.*, chorea-acanthocytosis/VPS13A disease (ChAc) and McLeod syndrome/XK disease (MLS), are respectively due to mutations in *VPS13A* and *XK* genes and share similar manifestations including the formation of acanthocytes. We previously showed by lipidomics of red blood cells (RBCs) from ChAc patients slight lipid changes compared to healthy controls. We here evaluated the consequences for RBC morphology, deformability, cytoskeleton and membrane lipid transversal and lateral distribution in five patients with ChAc and two patients with MLS. Compared to healthy donors, the two patient cohorts showed a strong increase of abnormal RBCs including acanthocytes and spheroechinocytes, a decrease in RBC projected surface area and deformability, and a rise in spectrin density. The abundance of cholesterol-enriched domains and the proportion of RBCs with ceramide-enriched patches were also increased while phosphatidylserine surface exposure was slightly decreased. In contrast, the abundance of sphingomyelin-enriched domains was poorly affected. At the individual level, patients showing the highest cholesterol-enriched domain abundance exhibited the highest number of RBCs with ceramide-enriched patches, compatible with RBC maturation defects, whereas patient RBCs exhibiting the highest spectrin membrane density showed the strongest loss of RBC projected surface area and the lowest abundance of sphingomyelin-enriched domains, consistent with RBC membrane alterations. Our study indicated that abnormal RBCs were associated with lipid distribution and cytoskeleton impairments, which appeared to result from both RBC maturation defects and membrane alterations. Moreover, the extent of lipid distribution alteration is well correlated with laboratory parameters typically altered in neuroacanthocytosis and could present an added value in neuroacanthocytosis syndrome evaluation.

## Introduction

Chorea-acanthocytosis (ChAc) or VPS13A disease (vacuolar protein sorting 13 homolog A) and McLeod syndrome (MLS) or XK disease are rare hereditary disorders (estimated prevalence 1:1,000,000 and 1:10,000,000 respectively) classified as neuroacanthocytosis syndromes because they are characterized by striatal neurodegeneration and the presence of acanthocytes (Walker et al. 2023; Peikert et al. 1993; Jung et al. 1993). ChAc and MLS share similar manifestations, including a variety of movement disorders, epilepsy, behavioral and cognitive impairment as well as peripheral neuropathy and myopathy (Peikert et al. 2002 [updated 2023]). Acanthocytes are red blood cells (RBCs) with few membrane protrusions varying in length and presenting a non-uniform distribution along the cell surface (Darras et al., 2021). In patients with ChAc those acanthocytes are associated with reduced deformability (Reichel et al., 2022; Recktenwald et al., 2022).

ChAc is an autosomal-recessive condition caused by pathogenic variants in the *VPS13A* gene, leading in most cases to a complete loss of the protein VPS13A/chorein (Dobson-Stone et al., 2004). VPS13A has been recently assigned to the superfamily of “bridge-like lipid transfer proteins,” which mediate direct lipid transfer between two organelles at membrane contact sites (Braschi et al. 2022; Kumar et al. 2018; Yeshaw et al. 2019; Hanna, Guillen-Samander, and De Camilli 2023). At the plasma membrane, it forms a complex with the XK protein, a putative phospholipid scramblase (Park and Neiman 2020; Guillén-Samander et al. 2022; Ryoden, Segawa, and Nagata 2022). MLS is an X-linked recessive disorder caused by mutations in the *XK* gene encoding for the XK protein and the Kx blood type antigen. Besides its interaction with VPS13A, the XK integral membrane protein forms a heterodimer with the Kell glycoprotein. XK absence has been described to lead to a reduction of phosphatidylserine (PS) in the inner plasma leaflet (Peikert, Hermann, and Danek 2022; Walker et al. 2023).

The mechanism behind formation of acanthocytes with reduced deformability in neuroacanthocytosis syndromes is poorly understood. We here tested the hypothesis that the RBC membrane lipid and spectrin distributions are impaired in those diseases. Indeed, as lipid transfer proteins contribute to the transport of lipids synthesized in the endoplasmic reticulum (ER) to other compartments, a mutation in *VPS13A* could greatly impact membrane lipid composition and distribution. Moreover, although mature RBCs do not contain an ER in contrast to nucleated cells or RBC precursors, they still present significant levels of VPS13A (Minetti et al., 2025). Supporting our hypothesis, elevated levels of several sphingolipids and phospholipids have been found in the striatum of VPS13 patients (Miltenberger-Miltenyi et al. 2023). Also, in Huntington’s disease, phenotypically very similar to ChAc and MLS diseases, a distinct shift in the sphingolipid profile of the caudate has been reported (Phillips et al. 2022). By lipidomic analysis of RBCs from five patients with ChAc, we recently revealed that phosphatidylethanolamine (PE) subspecies with long and more unsaturated acyl chains are increased while species with shorter and more saturated chains are decreased. A few ceramide (Cer) species are also increased in the disease (Peikert et al., 2024).

PE mainly associate with the plasma membrane inner leaflet and have been suggested to contribute to membrane-cytoskeleton interactions (Kapus and Janmey, 2013). The RBC cytoskeleton is composed of a meshwork of spectrin tetramers linked to the membrane by the 4.1R- and ankyrin-based anchorage complexes and is crucial for RBC deformation (Baines, 2010; Salomao et al., 2008). In ChAc RBCs, the linkage of Band3 to the 4.1R complexes is altered, contributing to the generation of acanthocytes (De Franceschi et al., 2011). This impairment appears to result from elevated tyrosine kinase Lyn activity, which hyperphosphorylates membrane proteins such as Band3 (De Franceschi et al., 2011). In addition, using RBCs from patients with ChAc and K562 erythroleukemic cells following *VPS13A* silencing, Foller and collaborators have reported reduced signaling via the PI3K-Rac1-PAK pathway (Foller et al., 2012).

Like PE, ceramides are also preferentially found in the inner plasma membrane leaflet and can cluster into Cer-enriched domains (Cloos et al., 2020). Three types of lipid domains have also been evidenced at the outer plasma membrane leaflet. The first ones are mainly enriched in cholesterol (named chol-enriched domains), associated with RBC high-curvature membrane areas and contribute to the RBC deformation process by gathering in the deformed area (Carquin et al., 2015; Carquin et al., 2016; Leonard et al., 2017a; Leonard et al, 2017b). The second and third ones associate with RBC low-curvature membrane areas and are respectively enriched in GM1 ganglioside, phosphatidylcholine (PC) and cholesterol (named GM1-enriched domains) and sphingomyelin (SM), PC and cholesterol (named SM-enriched domains) (Leonard et al., 2018; D'Auria et al., 2013; Conrard et al., 2018; Conrard and Tyteca, 2019; Stommen et al., 2023). Together with the spectrin cytoskeleton, GM1-enriched domains favor Ca^2+^ entry in RBCs through the mechanosensitive ion channel Piezo1 (Stommen et al., 2023).

To assess the impact of VPS13A or XK mutations on RBC morphology and deformability and the mechanism behind, we determined RBC morphology at rest and upon flowing, spectrin cytoskeleton organization, membrane transversal asymmetry as well as membrane lateral asymmetry in lipid domains using previously validated approaches (Leonard et al., 2017b; Cloos et al., 2020; Pollet et al., 2018; Pollet et al., 2020; Recktenwald et al., 2022; Carquin et al., 2015; Carquin et al., 2014). We also integrated data generated with those obtained for a patient suffering from another acanthocyte-related disease, the hypobetalipoproteinemia (Cloos et al., 2021). We finally discussed whether impairments could result from RBC maturation defects and/or alteration of the RBC membrane and how determination of lipid distribution could be useful in the evaluation of neuroacanthocytosis syndromes.

## Methods

### Blood collection and preparation

Five VPS13 patients, two MLS patients (Table 1), and five healthy controls (4 males and 1 female, 36–58 years) were included in this study. Among the five VPS13 patients, three (ChAc2, ChAc3, ChAc4) overlapped with the five patients included in our previous study (Peikert et al., 2024). Healthy donors were selected to be age- and gender-matched and their blood was processed in parallel to patients’ blood. The study was approved by the ethics committees at the Technische Universität Dresden (EK45022009, EK78022015), University Medical Center Rostock (A 2019-0134) and “Ärztekammer des Saarlandes” (ethics permission 51/18). All participants gave written informed consent in accordance with the Declaration of Helsinki. After collection, EDTA-coated tubes were transferred within 24 h to UCLouvain (Belgium) and Saarland University (Germany). For logistic reasons, not all patient samples were available at all investigation sites. ‘Incomplete’ datasets are therefore not a selection of patients for a particular assay.

**TABLE 1:** Overview of patients included in the study. M, Male. F, Female. PRN, pro re nata. N/A, not applicable.

Patient	Sex	Age (years)	Main clinical manifestation	Disease duration (years)^a^	Chorein Western blot	Medications	Nutritional lifestyle
ChAc1	M	41	Epilepsy, chorea, vocal tics, peripheral neuropathy, cognitive impairment	3	Chorein band weak	Levetiracetam 3,000 mg/d Sertralin 100 mg/d	Varied, well-balanced meals, obesity
ChAc2	F	54	Epilepsy, parkinsonism, dystonia, dysarthria peripheral neuropathy, cognitive impairment	33	Chorein absent	Levetiracetam 4,000 mg/d Valproate 2,000 mg/d Clobazam 10 mg/d Zonisamide 200 mg/d	Varied, well-balanced meals, Vitamin D supplementation
ChAc3	M	56	Parkinsonism, dystonia, dysarthria, peripheral neuropathy mild depression	18	Chorein absent	Scopoderm transdermal therapeutic system/day, PRN: Melperone 25 mg	Varied, well-balanced meals
ChAc4	M	37	Drug resistant epilepsy, mild chorea, tics, cognitive impairment, peripheral neuropathy, myopathy	14	Chorein absent	Lacosamide 550 mg/d Zonisamide 300 mg/d Perampanel 4 mg PRN: Lorazepam/Midazolam	Varied, well-balanced meals, Vitamin D and folate supplementation
ChAc5	M	32	Drug resistant epilepsy, mild chorea, tics, cognitive impairment, irritability, anxiety, depression, psychosis	18	Chorein absent	Lacosamide 600 mg/d Zonisamide 50 mg/d Cenobamat 200 mg/d Mirtazapine 7.5 mg/d Aripiprazole 15 mg/d	Varied, well-balanced meals, Vitamin D supplementation
MLS1	M	57	Cardiomyopathy, peripheral neuropathy, myopathy	6	N/A	Bisoprolol 5 mg/d Eplerenon 50 mg/d Torasemid 5 mg/d Apixaban 10 mg/d	Varied, well-balanced meals, obesity
MLS2	M	54	Epilepsy, peripheral neuropathy, myopathy	43	N/A	Levetiracetam 2,000 mg/d Lamotrigin 400 mg/d Candesartan 8 mg/d	Varied, well-balanced meals, Vitamin D supplementation

^a^ Since onset of first symptoms

All experiments (except blood parameters and microfluidic) were performed on RBCs separated from other blood components through 10-fold blood dilution in a high glucose- and HEPES-containing medium (Dulbecco’s modified eagle medium [DMEM]). Diluted blood was centrifugated at 200 ×*g* for 2 min, the supernatant removed and RBCs resuspended in medium. RBCs were then washed a second time in the same conditions, as in (Cloos et al., 2020). For the microfluidic measurements, blood samples were suspended in phosphate-buffered saline (PBS) solution and centrifuged for 5 min at 1,500 × g for 5 min to separate the RBCs from plasma and most leukocytes and platelets. Subsequently, sedimented RBCs were resuspended in PBS, and the centrifugation and washing steps were repeated three times. Finally, a hematocrit of 0.5% was adjusted in a PBS solution that contained 1 g/L bovine serum albumin (BSA).

### Blood parameters

The blood hemoglobin concentration, hematocrit, erythrocyte number, reticulocyte count, mean corpuscular volume (MCV), mean corpuscular hemoglobin (MCH) and concentration (MCHC), hepatic enzymes as well as plasma cholesterol and triglyceride contents were determined at the University Hospital Carl Gustav Carus Dresden and the University Medical Center Rostock during the patients’ yearly medical appointments.

### RBC morphology determination

Washed RBCs were analyzed in suspension in µ-dish ibidi chambers, after a 24-fold dilution in DMEM, by vital optical microscopy (Observer.Z1; plan-Apochromat 100 × 1.4 oil Ph3 objective), as in (Cloos et al., 2021). The respective abundance of discocytes, stomatocytes and abnormal RBCs (*i.e.*, acanthocytes, echinocytes, spheroechinocytes and elliptocytes) was manually counted on at least 10 images per ibidi chamber and expressed as % of the total population and then in % difference of the corresponding healthy donor. Acanthocytes were defined as abnormal RBCs with spikes of different lengths and widths unevenly positioned at the cell surface, echinocytes as RBCs with numerous fine and uniform spicules along the periphery, spheroechinocytes as spherical cells with diameter smaller than 7 µm and whose spicules have become fine needle-like projections, and elliptocytes as elongated oval-shaped RBCs.

### Determination of projected surface area of RBCs

This parameter was determined by optical microscopy on living washed RBCs spread on coverslips coated with 0.01% poly-L-lysine (PLL; Sigma-Aldrich). Although this procedure induces a loss of RBC biconcavity, it does not affect RBC viability. Briefly, coverslips were coated with PLL for 30 min at 37°C and washed 2 times with DMEM. Then, washed RBCs were spread for 4 min on the coverslips which were thereafter rinsed to remove unattached RBCs, placed upside down in LabTek chambers filled with DMEM and observed with the microscope Observer. Z1. The hemi-RBC area was determined by manually surrounding the RBC surface using the ImageJ software and expressed as % of RBC area of healthy donors.

### Microfluidic measurements

Blood samples were measured within 2–8 h from the withdrawal. The Erysense® device (Cysmic, Saarbrücken, Germany) was used as previously described (Recktenwald et al., 2022). In short, it employs a microfluidic chip with parallel microfluidic channels that have a rectangular cross-section with a height of 8 µm, a width of 11 µm, and a total length of 40 mm. A constant pressure drop in a range of 100 mbar to 1 bar is used to pump the RBC suspension through the microfluidic chip. RBC flow is recorded with a frame rate of up to 400 Hz depending on the applied pressure drop and subsequently processed. To enable a fast and automated classification of RBC shapes, we used a convolutional neural network (CNN), as described previously (Kihm et al., 2018). The CNN consists of an image input layer, several subsequent convolution stages, and an output layer. We employed a supervised training of the CNN according to (Kihm et al., 2018). Our training data set consisted of seven different classes. Besides the characteristic croissant and slipper shapes that dominate healthy RBC flow in microchannels (Kihm et al., 2018), complimentary pathological classes that exhibit pathophysiologic RBC shapes were identified. A summary of the RBC shapes for a given sample is the so-called RBC shape phase diagram, *i.e.*, the frequency of occurrence of RBC shapes as a function of their velocity.

### Spectrin immunofluorescence

Immunolabelling of spectrin was performed as in (Cloos et al., 2020; Pollet et al., 2020; Stommen et al., 2023). Briefly, washed RBCs were diluted 12-fold in DMEM, immobilized onto PLL/PBS (1:1)-coated coverslips for 4 min, washed and permeabilized with PBS/0.5% Triton X-100 under agitation for 3 min to open the RBCs and have access to the cytoskeleton overhanging the PLL-coated RBC membrane. After 3 new washes with PBS, RBCs were fixed with 4% (v/v) paraformaldehyde under agitation for 10 min, rinsed 3 times with PBS and blocked with PBS/3% BSA (w/v) under agitation for 1 h. Next, coverslips were incubated with antibodies against α/β-spectrin (Merck) diluted in PBS/0.2% BSA for 1h30, washed 3 times in PBS/3% BSA, incubated with Alexa-secondary antibodies diluted in PBS 0.2% BSA, in the dark for 1 h and finally washed again 2 times with PBS/3% BSA and one last time with PBS. All steps were performed at room temperature (RT). Coverslips were mounted with Dako and examined with a Zeiss LSM980 confocal microscope using a plan-Apochromat 63x NA 1.4 oil immersion objective and the same settings for illumination inside one experiment. RBC membrane spectrin occupancy was then determined with the ImageJ software.

The acquired Zeiss images were first imported in ImageJ and segmented. The best threshold value was determined by first analysing the images by the “automatic threshold” option proposed by the software. Results were then visually inspected and parameters recorded. In a second round, the selected value was fixed and used for the patient and corresponding control images. However, due to biological and experimental variability over time (e.g., PLL coating, laser conditions, antibody lots), this fixed value for segmentation could not be successfully used for all experiments and was therefore slightly adapted in one experiment to another but was always the same for the patient and its internal control inside one experiment. Then, using the determined threshold, 10 images/condition were quantified. For all images, a maximum number of individual RBCs per image was circled (regions of interest, ROI) while avoiding blurred, pressed or superimposed RBCs showing very intense central “lines” (suggestive of membrane folds), to minimize artifacts (Supplementary Figure 1). Images were transformed to a binary image, where the selected (white) pixels correspond to the spectrin labelling. This analysis provides a qualitative estimation of the spectrin attachment to the membrane. These pixels do not correspond to a real % of spectrin occupancy, as it depends on the threshold used, but it allows conditions to be compared. Spectrin occupancy % in the surrounded RBCs (all images combined) for the patient was finally expressed as % of the value obtained in corresponding healthy donor.

### Membrane transversal asymmetry

Exposure of PS was analyzed by flow cytometry as in (Cloos et al., 2020). Briefly, washed living RBCs were diluted 10-fold in DMEM and then 12.5-fold in DMEM containing Annexin V FITC (5-fold dilution, Invitrogen). The labeling was performed in suspension at RT for 20 min. To determine the % of PS-exposed RBCs in the patients, the intensity bar (FITC) was positioned at the limit of the overall RBC population of the corresponding healthy control. This cursor was then positioned at the same location on the patient population. From there, the proportion of RBCs that stand out from the general population was determined by the Flow Jo program.

### Membrane lipid imaging

Membrane lipids were visualized by fluorescence microscopy on washed living RBCs. To label cholesterol, RBCs were diluted 12-fold in DMEM containing 0.1% BSA free fatty acids and Theta toxin fragment (0.45–1.2 µM, depending on production and purification) and incubated in suspension at RT for 20 min, pelleted and resuspended in DMEM and finally immobilized onto DMEM/PLL-coated coverslips for 4 min (Cloos et al., 2020; Cloos et al., 2023). To visualize SM and ceramide, washed RBCs were first spread onto DMEM/PLL-coated coverslips and then labelled by trace insertion in the plasma membrane of BODIPY fluorescent analogs of these sphingolipids at RT for 20 min as described in (Conrard et al., 2018). Coverslips with labelled immobilized RBCs were then placed in DMEM-filled LabTek chambers and observed with the Observer. Z1 (plan-Apochromat 100 × 1.4 oil Ph3 objective). Abundance of chol- and SM-enriched domains per RBC was manually counted and normalized on the RBC hemi-area, calculated with the ImageJ software. The proportion of RBCs presenting Cer-enriched patches was also assessed by manual counting. All data were finally expressed as % of healthy RBCs.

### Data presentation and statistical analyses

Each patient was associated to an acronym (ChAc for Chorea-acanthocytosis; MLS for McLeod syndrome), a number according to its order of inclusion into the study and a color code according to the syndrome (red for ChAc; green for MLS). Data are depicted as means ± SD. Three types of statistical analyses were performed. First, the comparison of the global patient cohort with the healthy donors was done by parametric one sample t-test or non-parametric Wilcoxon signed-rank test. Second, the comparison of the ChAc patient cohort with the corresponding healthy donors was also done by parametric one sample t-test or non-parametric Wilcoxon signed-rank test. However, we were not able to compare the MLS patient cohort with the healthy donors or the two disease groups together because the MLS group contained only 2 patients. Third, patients were individually compared to their respective shipment healthy donor, if at least 3 independent experiments were performed for one patient (non-parametric Kruskal-Wallis test and Dunn’s multiple comparison test). For correlations, linear Pearson regressions were plotted on the graphs only when the coefficient of determination (r^2^) was higher than 0.45.

### Calculation of scores for laboratory parameters, abnormal RBC proportion and lipid distribution

**FIGURE 1:**
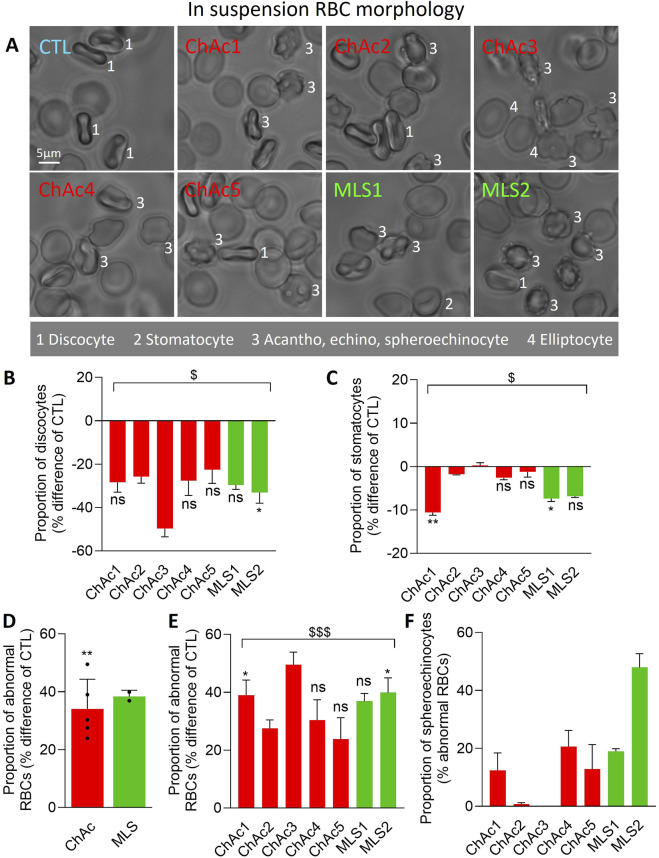
Patients show a high but differential proportion and type of abnormal RBCs. Chorea-acanthocytosis (ChAc; red) and McLeod syndrome (MLS; green) patients were compared to healthy donors (CTL) for morphology of RBCs in suspension. **(A)** Representative images. 1, discocytes; 2, stomatocytes; 3, acanthocytes, echinocytes, spheroechinocytes; 4, elliptocytes. **(B–E)** Quantification of the relative abundance of discocytes **(B)**, stomatocytes **(C)** and abnormal RBCs, *i.e.*, populations 3 & 4 in A **(D, E)**, expressed as % of the global RBC population for each patient and then in % difference of the corresponding healthy donor. **(F)** Proportion of spheroechinocytes expressed as % of all abnormal RBCs (acanthocytes, echinocytes and spheroechinocytes). Data are presented either per individual patient [means ± SD of 3 independent experiments, except for ChAc2 and ChAc3; **(B, C, E, F)**] or per patient cohort [means ± SD of 5 patients for ChAc or 2 patients for MLS; **(D)**]. Statistical analyses are indicated (i) next to the bar (top or bottom) for the comparison of individual patients vs. their corresponding healthy donors [**(B, C, E)**; Kruskal–Wallis test with Dunn’s multiple comparisons test; ns, not significant; *, p < 0.05; **, p < 0.01)]; (ii) above the ChAc bar for the whole ChAc cohort vs. the corresponding healthy cohort [**(D)**; one sample t-test; ^**^, p < 0.01)]; and (iii) above a bracket for the 7 patients together vs*.* the corresponding group of healthy donors [**(B, C, E)**; one sample t-test; ^$^, p < 0.05; ^$$$^, p < 0.001)].

To determine the laboratory parameters score, the number of out-of-range values per patient depicted in red in Table 2 were added. The obtained value was then divided by the number of parameters provided in this Table for each patient: a higher score was therefore attributed to a more affected patient, in the range from 0 to 5 (0 = no alteration; 5 = strongest alteration). The abnormal RBC proportion score was assessed from Figure 1E: a higher score was attributed to a higher number of abnormal RBCs, in the range from 0 to 5 (0 = no change; 5 = 50% abnormal RBCs in % difference of CTL). Finally, the lipid distribution score was obtained by adding three scores, one for chol-enriched domains from Figure 6C, one for SM-enriched domains from Figure 6F and one for Cer-enriched patches from Figure 7C (e.g., for chol-enriched domains: 1 = no change; 4.5 = 450% of healthy RBCs): the higher the score, the higher the increase of domains/patches as compared to healthy RBCs.

**TABLE 2:** Laboratory parameters of patients included in the study. MCV, mean corpuscular volume; MCH, mean corpuscular hemoglobin; MCHC, mean corpuscular hemoglobin concentration; ASAT, Aspartate aminotransferase; ALAT, Alanine aminotransferase; CK, Creatine kinase; LDH, lactate dehydrogenase. Values outside the reference range are indicated in red. ND, not determined. µmol/s*L, amount of substrate converted by the enzyme in moles per unit time and volume.

Parameter (Units)	ChAc1	ChAc2	ChAc3	ChAc4	ChAc5	MLS1	MLS2	Normal range
Hemoglobin (mmol/L)	9.6	8.7	9.3	9.4	9.9	9.8	9.4	7.4–12
Hematocrit	0.45	0.39	0.41	0.44	0.46	0.46	0.44	0.37–0.47
Erythrocytes (* 10^12^/L)	5.53	4.21	4.66	5.07	5.03	5.25	4.65	4.4–5.77
Reticulocytes (* 10^10^/L)	21.2	ND	ND	9.33	9.86	10.9	9.9	2–14
MCV (fL)	81.2	93	87	85.8	91.5	87.8	93.5	81–96
MCH (fmol)	1.74	2.07	2	1.85	1.97	1.87	2.02	1.7–2.2
MCHC (mmol/L)	21.4	22.3	22.9	21.6	21.5	21.3	21.6	18.5–22.5
ASAT (U/L; or µmol/s*L)	52.1	0.35	0.55	103	99.5	72.5	72.2	< 50 < 0.60
ALAT (U/L; or µmol/s*L)	68.6	0.21	0.57	118	125	69.7	76.4	< 50 < 0.60
CK (U/L)	1267	109	511	3409	2538	1855	3134	< 190
LDH (U/L; or µmol/s*L)	285	3.97	6.61	441	406	429	477	< 250 2.25–3.55
Cholesterol (mmol/L)	5.6	4.51	ND	4.5	5.2	3.4	5.4	< 6.18
HDL-cholesterol (mmol/L)	1.24	ND	ND	0.97	1.26	0.92	0.96	0.91–2.06
LDL-cholesterol (mmol/L)	3.71	ND	1.49	3.01	3.39	2.14	3.43	1.76–4.11
Triglycerides (mmol/L)	3.73	3.27	ND	1.9	1.59	1.45	4.67	< 1.7

### Data sharing statement

For original data, please contact the corresponding author.

## Results

### Clinical and blood parameters of the 7 patients included in the study

Five ChAc and two MLS patients were included in this study. Six of them are men and were, at the time of the study, between 32 and 57 years old (Table 1, 3 first columns). The diagnosis of ChAc and MLS has been proven by genetic testing, and additionally for all ChAc patients, by chorein Western blot (Table 1, sixth column). The clinical manifestations are typical and reflect the known broad interindividual heterogeneity (Table 1, fourth column). None of the patients have a particular nutritional lifestyle (Table 1, eighth column). Regarding blood parameters, aspartate aminotransferase (ASAT), alanine aminotransferase (ALAT) and creatine kinase (CK) are out of normal ranges (highlighted in red in Table 2), except for ChAc2 and ChAc3. Conversely, lactate dehydrogenase (LDH) is increased in all patients. While plasma cholesterol levels are in the normal ranges, triglycerides are increased in all patients, except ChAc5 and MLS1. Hemoglobin levels, hematocrit, erythrocyte and reticulocyte counts, RBC mean corpuscular volume (MCV) and hemoglobin concentration (MCHC) are within the normal range in almost all patients. The only exceptions are a higher reticulocyte level in ChAc1 (21.2 vs. 2–14*10^10^/L in normal subjects; Table 2) and a higher MCHC in ChAc3 (22.9 vs. 18.5–22.5 mmol/L in normal subjects; Table 2). Thus, based on laboratory parameters, ChAc1 appears the most affected patient. Surprisingly, he was the only one with a weak chorein (VPS13A) band in Western blot whereas the other patients showed no chorein band (Table 1, sixth column). Thus, there is an apparent discrepancy between blood parameters and diagnosis based on chorein content determined by Western blotting, which prompted us to further compare the 7 patients for RBC morphology, cytoskeleton and lipid distribution.

### Patients exhibit a high proportion of abnormal RBCs, including spheroechinocytes in variable proportion, and a lower projected surface area of RBCs

**FIGURE 2:**
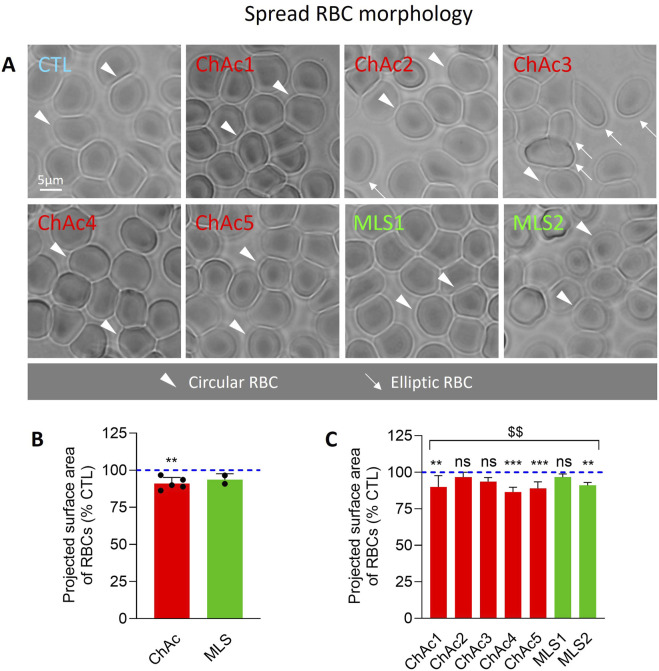
Patient RBCs show a decreased projected area. RBCs from chorea-acanthocytosis (ChAc; red) and McLeod syndrome (MLS; green) patients were compared to RBCs from healthy donors (CTL) for projected surface area after spreading on poly-L-lysine (PLL). **(A)** Representative light microscopy images of living RBCs spread on PLL-coated coverslips. Arrowheads, circular RBCs; arrows, elliptic RBCs. **(B, C)** Quantification of the projected surface area of RBCs. Data are expressed as % of healthy donors and presented either per patient cohort [means ± SD of 5 or 2 patients; **(B)**] or per individual patient [means ± SD of 9–10 independent experiments; **(C)**]. Statistical analyses are indicated (i) next to the ChAc bar for the comparison of the whole ChAc cohort vs. the corresponding healthy cohort [**(B)**; one sample t-test; ^**^, p < 0.01)]; (ii) next to the concerned bar for individual patients vs. corresponding healthy donors [**(C)**; Kruskal–Wallis test with Dunn’s multiple comparisons test; ns, not significant; **, p < 0.01; ***, p < 0.001)]; and (iii) above a bracket for the 7 patients together vs*.* the corresponding group of healthy donors [**(C)**; one sample t-test; ^$$^, p < 0.01)].

We started by evaluating by optical microscopy of living RBCs in suspension the extent of morphology alterations (Figure 1A). The proportion of discocytes and to a lower extent of stomatocytes was decreased in the overall cohort of patients (Figures 1A–C) at the benefit of abnormal RBCs, *i.e.*, acanthocytes, echinocytes, spheroechinocytes and elliptocytes (defined in Material and Method; Figures 1A, D, E). The lowest increase was seen in ChAc5 and the highest increase in ChAc3 (Figure 1E). Among those abnormal RBCs, spheroechinocytes were particularly abundant in MLS2 but absent in ChAc2 and ChAc3 (Figure 1F) and elliptocytes were mainly detected in ChAc3 (Figures 1A, 2A). The remaining abnormal RBCs were acanthocytes and echinocytes, which represented the large majority of these abnormal RBCs, as expected (Figure 1A). Besides changes in RBC morphology, optical microscopy of RBCs spread on PLL-coated coverslips revealed a slight decrease of the projected surface area of RBCs in both diseases (Figures 2A, B). This decrease was however non-significant in ChAc2, ChAc3 and MLS1 (Figure 2C). Altogether, we showed a strong increase of abnormal RBCs, including spheroechinocytes in variable proportion from one patient to another. Moreover, patients showing the greatest increase in spheroechinocytes accordingly exhibited a significant decrease in the projected surface area of RBCs. Such variability from one patient to another could suggest differential RBC deformability and spectrin cytoskeleton impairment.

### Patient RBCs exhibit increased proportion of RBCs with higher rigidity and lower deformability upon flowing

**FIGURE 3:**
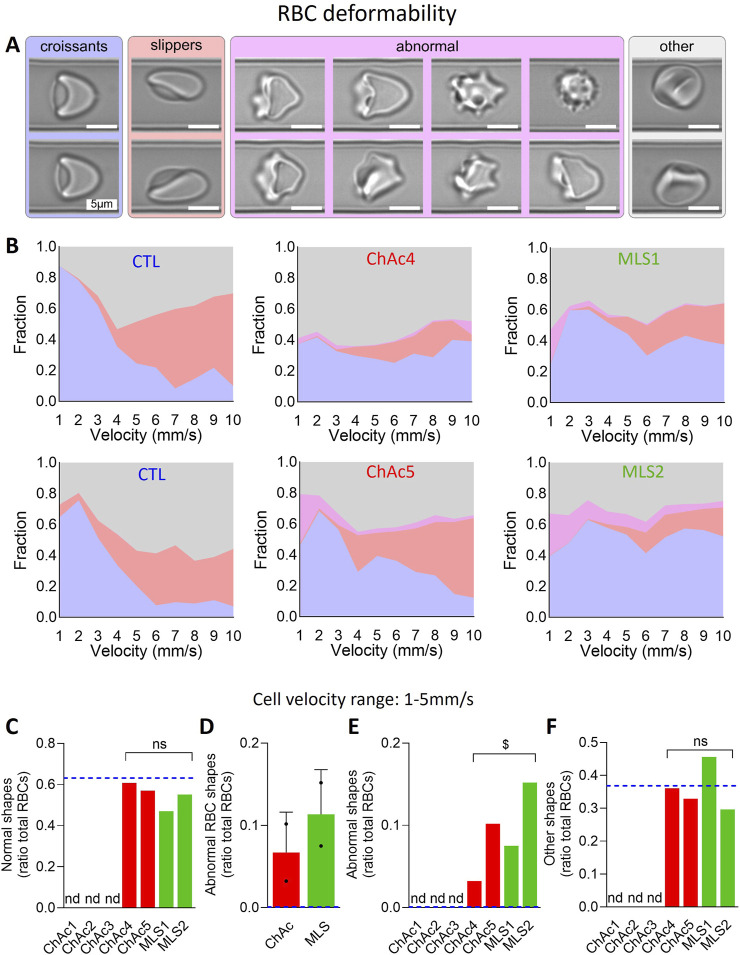
Patients show a higher but differential proportion of abnormal RBCs upon microcapillary flow. RBCs from chorea-acanthocytosis (ChAc; red) and McLeod syndrome (MLS; green) patients were compared to RBCs from healthy donors [CTL; blue dotted lines in **(C–F)**] for morphology upon deformation during low shear stress flow in an 11 μm × 9 µm channel. **(A)** Examples of RBC shapes. Blue, croissant-like RBCs; red, slippers; pink, abnormal RBCs which include echinocytes, acanthocytes and spheroechinocytes; grey, others, *i.e.*, not a well-defined cell type but which refers to cells that the AI-based algorithm could not classify in one of the aforementioned cell classes. **(B)** Fraction of each RBC class in a deformed state at a given cell velocity in the channel for 2 representative healthy donors (among 4), 2 ChAc and the 2 MLS patients. **(C–F)** Fraction of normal RBCs [**(C)**; *i.e.*, croissants and slippers shown in **(A, B)**], abnormal RBCs **(D, E)** and RBCs with other shapes **(F)** in the cell velocity range of 1–5 mm/s. Data are expressed as ratio of total RBCs and compared to CTL samples (blue dotted lines) and presented either per individual patient **(C, E, F)** or per patient cohort [means ± SD of 2 patients each; **(D)**]. Nd, not determined. Statistical analyses for the 4 patients together vs*.* the corresponding group of healthy donors are indicated above a bracket [**(C, E, F)**; one sample t-test; ns, not significant; ^$^, p < 0.05].

To test the above hypothesis, we first determined the RBC morphology upon flowing through a channel with a width of 11 μm and height of 9 μm at cell velocities ranging from 1 to 10 mm/s. RBCs were classified as normal (*i.e.*, croissants, slippers), abnormal (*i.e.*, echinocytes, acanthocytes, spheroechinocytes) or other (*i.e.*, different from the other classes; Figure 3A). Among the four patients tested, MLS2, who showed the highest increase in spheroechinocytes (Figure 1F), also exhibited the highest increase of abnormal RBC shapes upon flowing (Figure 3B). To test whether this observation could reflect an increased RBC rigidity as previously suggested (Rabe et al., 2021), we determined the fraction of each RBC population in the cell velocity range of 1–5 mm/s (Figures 3C–F). We revealed a significant increase in abnormal and other RBC populations, which together corresponded to the proportion of abnormal RBCs measured in ibidi chambers (Figures 3E, F vs*.* Figure 1E). Altogether, patients have a significant increase in abnormal RBCs also under microfluidic flow conditions and lower RBC deformability.

### Patient RBCs show a denser spectrin cytoskeleton

**FIGURE 4:**
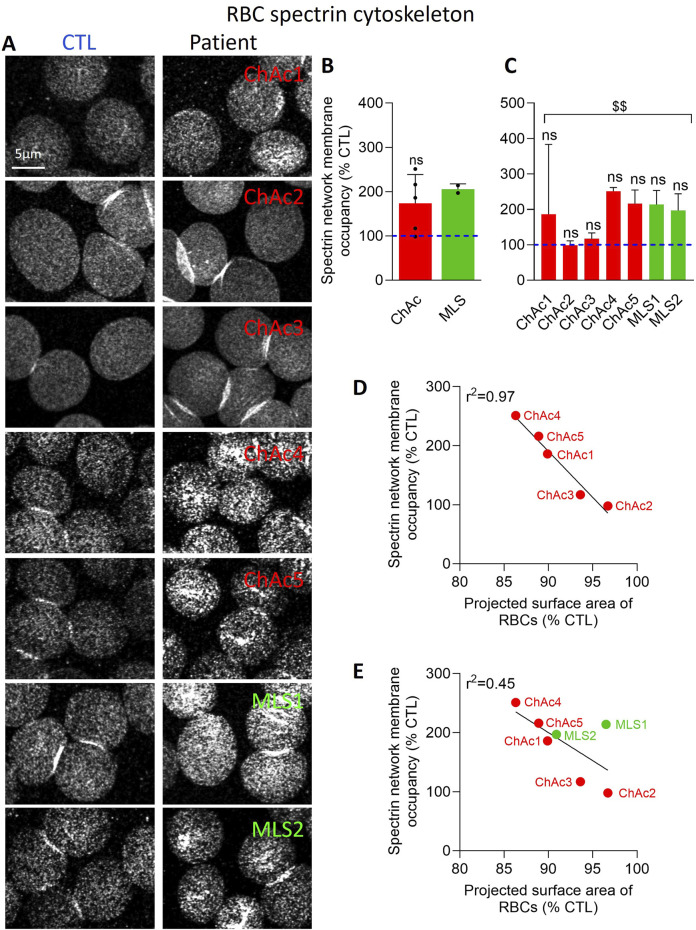
The spectrin network density is increased in the global patient cohort and correlates inversely with the projected surface area of RBCs. RBCs from healthy donors (CTL, blue dotted lines) or ChAc and MLS patients (red and green, respectively) were spread onto PLL-coated coverslips, permeabilized, fixed, immunolabelled for spectrin and visualized by confocal microscopy using the same settings for sample illumination inside one experiment. **(A)** Representative images of patient RBCs compared to their corresponding healthy donor. **(B, C)** Quantification of spectrin occupancy. Data are expressed as % of healthy RBCs and presented either per patient cohort [means ± SD of 5 or 2 patients; **(B)**] or per individual patient [means ± SD of 3 independent experiments; **(C)**]. Statistical analyses are indicated (i) next to the ChAc bar for the comparison of the whole ChAc cohort vs. the corresponding healthy cohort [**(B)**; one sample t-test; ns, not significant]; (ii) next to the concerned bar for individual patients vs. corresponding healthy donors [**(C)**; Kruskal-Wallis test with Dunn’s multiple comparisons test; ns, not significant]; and (iii) above a bracket for the 7 patients together vs*.* the corresponding group of healthy donors [**(C)**; one sample t-test; ^$$^, p < 0.01]. **(D, E)** Correlations between the projected surface area of RBCs (from Figure 2C) and spectrin network membrane occupancy [from **(4C)**] taking into account either the 5 ChAc patients only **(D)** or all the 7 patients **(E)**.

We then evaluated the spectrin cytoskeleton of spread RBCs permeabilized before fixation to have access to the cytoskeleton overhanging the PLL-coated RBC membrane. This procedure has allowed us in the past to reveal a homogeneous spectrin network in healthy RBCs by both confocal and ‘Airyscan’ microscopy (Stommen et al., 2023). In Airyscan microscopy, physical pinhole and the unitary Airyscan detector use a new pinhole plane image detection approach based on a 32-channel GaAsP-PMT area detector. Each of the 32 detector elements acts as its own small pinhole with positional information. The new positional information allows for increased contrast of high-spatial frequency information previously not available in traditional confocal systems. Ultimately, Airyscan resolution is 120 nm in the focal plane. Fixation in suspension before permeabilization generated similar data (Stommen et al., 2023) and RBC storage at 4°C was used as positive control as it increases the spectrin membrane occupancy (Cloos et al., 2020). In both diseases, the spectrin network occupancy per RBC surface increased, but it was not significant (Figure 4B). A significant effect was only reached upon pooling the 7 patients together (Figure 4C). However, analysis of ChAc patients individually indicated that ChAc2 and ChAc3, who did not exhibit any spheroechinocyte and changes in the projected surface area of RBCs, presented a spectrin network membrane occupancy similar to healthy RBCs. In contrast, ChAc4, who presented the strongest decrease in the projected surface area of RBCs, exhibited the highest increase of the spectrin network density (Figures 4A–C). These data suggested a relation between the spectrin network membrane occupancy and the projected surface area of RBCs in ChAc, as confirmed by the excellent inverse correlations between these parameters (Figure 4D). Nevertheless, this correlation was strongly decreased when taking into account the two MLS patients (Figure 4E), which could suggest a differential impact of the two diseases on the RBC cytoskeleton.

### Patients show a slightly reduced proportion of RBCs exhibiting phosphatidylserine surface exposure

**FIGURE 5:**
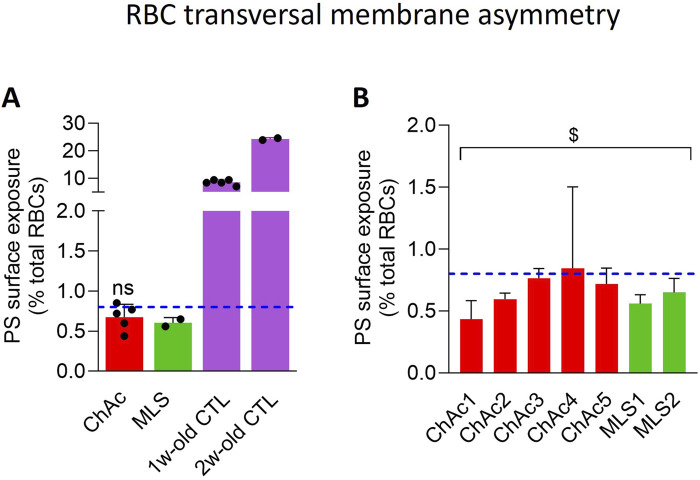
The PS surface exposure is slightly but significantly decreased in the global patient cohort. RBCs from healthy donors (CTL, blue dotted line) or ChAc and MLS patients (red and green, respectively) were labelled with fluorescent Annexin-V and analyzed with FlowJo to determine the proportion of PS-exposing cells by positioning the cursor at the edge of the healthy fresh RBC population. RBCs from blood stored for 1 or 2 weeks at 4°C were used as positive control [mauve dotted columns in **(A)**]. Data are expressed as % of total RBCs and presented either per patient cohort [means ± SD of 5 or 2 patients; **(A)**] or per individual patient [means ± SD of triplicates; **(B)**]. Statistical analyses are indicated (i) next to the ChAc bar for the comparison of the whole ChAc cohort vs. the corresponding healthy cohort [**(A)**; one sample t-test; ns, not significant]; and (ii) above a bracket for the 7 patients together vs*.* the corresponding group of healthy donors [**(B)**; one sample t-test; ^$^, p < 0.05].

To next explore whether the lower RBC area and higher spectrin network density could be associated with an impairment of membrane lipid transversal asymmetry, we determined the extent of PS exposure at the RBC surface through labelling with fluorescent Annexin V (Ghodsi et al., 2023). As expected, the proportion of healthy RBCs showing PS surface exposure was lower than 1% in fresh state and increased to ∼10 and ∼25% upon blood storage for 1 and 2 weeks at 4°C, used as positive controls for the technique. In contrast, in the two patient cohorts, the PS exposure showed a tendency to decrease as compared to healthy fresh RBCs, but it was not significant (Figure 5A). A significant effect was only reached upon pooling the 7 patients together (Figure 5B). These data indicated that the PS exposure was not exacerbated in the patient RBCs, quite the contrary.

### Patient RBCs exhibit a higher abundance of cholesterol-enriched domains, which inversely correlates with the level of abnormal RBCs

**FIGURE 6:**
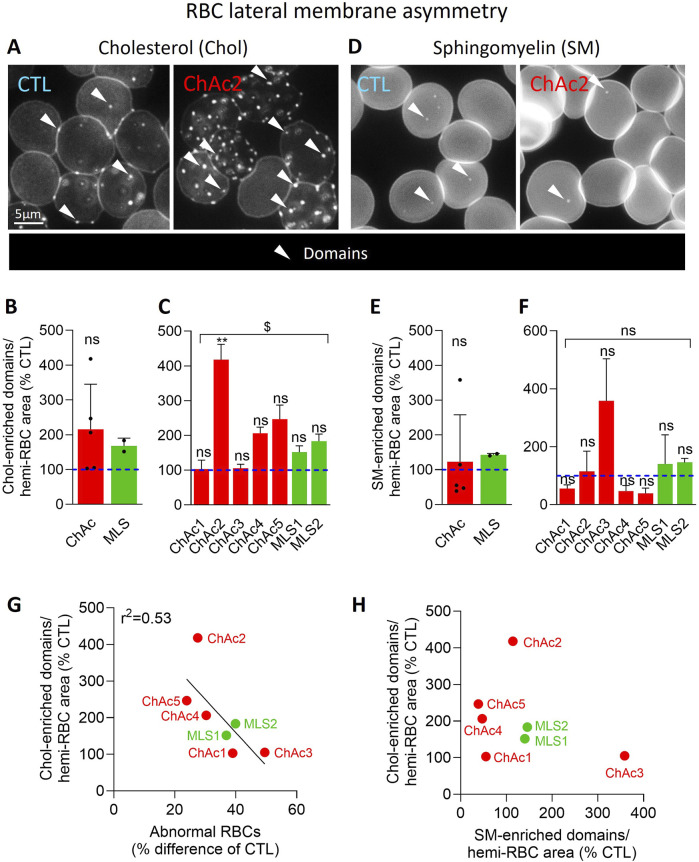
In contrast to SM-enriched domains, chol-enriched domains are increased in the global patient cohort and correlates inversely with the proportion of abnormal RBCs. RBCs from healthy donors (CTL, blue) or ChAc and MLS patients (red and green, respectively) were either labelled with the mCherry-Theta toxin fragment specific to endogenous cholesterol and then immobilized on PLL-coated coverslips **(A–C)**; or immobilized on PLL-coated coverslips and then labelled with fluorescent BODIPY-SM **(D–F)**. All coverslips were then directly observed by vital fluorescence microscopy. **(A, D)** Representative images of chol- and SM-enriched domains in a healthy donor and a patient. Arrowheads, lipid-enriched domains. **(B, C, E, F)** Quantification of lipid domain abundance normalized to the hemi-RBC area. Data are expressed in % of healthy donors and presented either per patient cohort [means ± SD of 5 or 2 patients; **(B, E)**] or per individual patient [means ± SD of 3 independent experiments; **(C, F)**]. Statistical analyses are indicated (i) next to the ChAc bar for the comparison of the whole ChAc cohort vs. the corresponding healthy cohort [**(B)**, one sample t-test; **(E)**, Wilcoxon signed-rank test; ns, not significant]; (ii) next to the concerned bar for individual patients vs. corresponding healthy donors [**(C, F)**; Kruskal–Wallis test with Dunn’s multiple comparisons test; ns, not significant; **, p < 0.01]; and (iii) above a bracket for the 7 patients together vs*.*the corresponding group of healthy donors [**(C)**, one sample t-test; **(F)**, Wilcoxon signed-rank test; ns, not significant; ^$^, p < 0.05]. **(G)** Correlation between abnormal RBC proportion [data from Figure 1E] and chol-enriched domain abundance [Data from **(C)**]. **(H)** Absence of correlation between chol- and SM-enriched domain abundance [data from **(C, F)**, respectively].

We then analyzed the cholesterol lateral distribution in submicrometric domains at the RBC outer plasma membrane leaflet through labelling with the mCherry-Theta toxin fragment (Carquin et al., 2015). In both diseases, the abundance of chol-enriched domains increased by ∼2-fold in the patient cohort vs*.* healthy donors (Figure 6B). This increase was visible in all patients except ChAc1 and ChAc3 but was only significant in ChAc2 compared to the respective healthy controls (Figures 6A, C) and was stronger in patients showing the lowest increase of abnormal RBCs (Figure 6G; Supplementary Figure 2A).

### Patient RBCs present differential alterations of sphingomyelin-enriched domain abundance, which do not correlate with the alterations of cholesterol-enriched domains

To assess whether this observation could be extended to another type of domains, RBCs were labelled with a BODIPY-SM analog to identify SM-enriched domains. In contrast to chol-enriched domains, the abundance of SM-enriched domains was not significantly altered in either ChAc or MLS but showed a high variability which was due to sometimes opposite effects in individual patients (Figures 6E, F). For instance, while ChAc3 showed a strong increase, ChAc1, 4 and 5 exhibited a reduction of SM-enriched domains. Furthermore, SM-enriched domain changes did not correlate with the abundance of chol-enriched domains (Figure 6H), suggesting that both domain alterations contributed to the disease in a different manner.

### Patients show an increased abundance of RBCs with ceramide-enriched patches, which positively correlates with cholesterol- but not sphingomyelin-enriched domains

**FIGURE 7:**
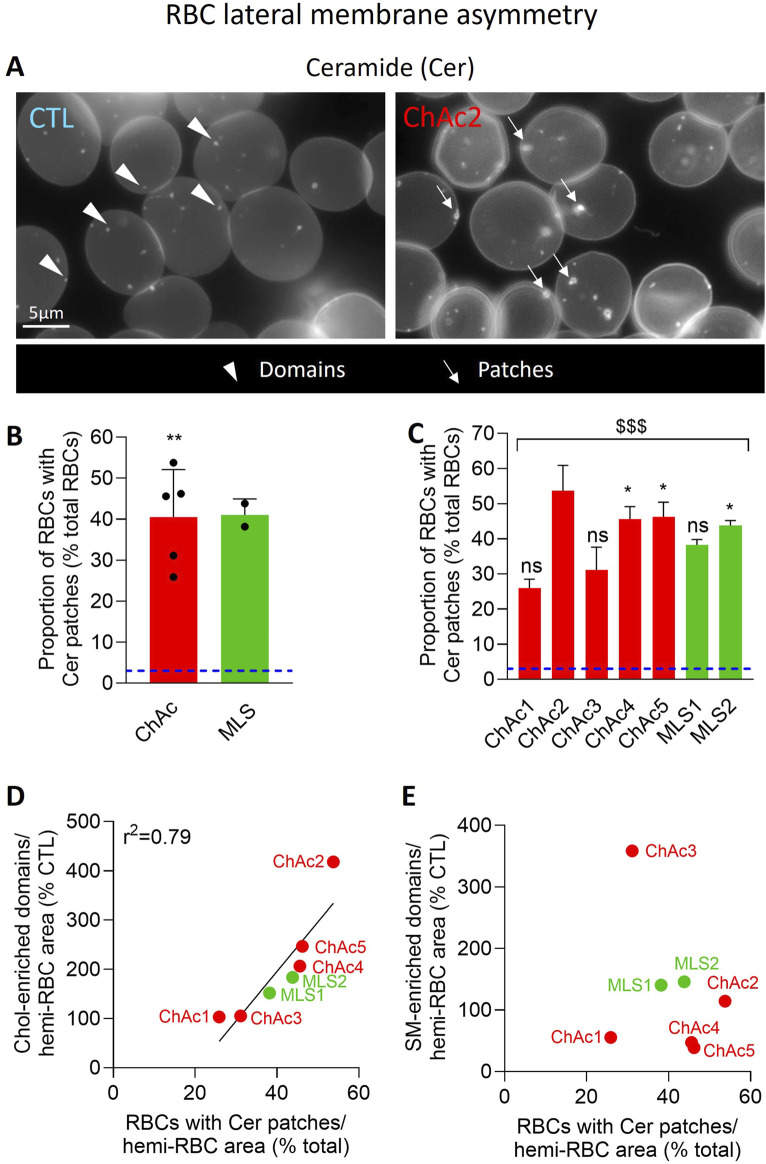
The proportion of RBCs with Cer-enriched patches is highly increased in the global patient cohort. RBCs from healthy donors [blue dotted line at **(B, C)**] or ChAc and MLS patients (red and green, respectively) were immobilized on PLL-coated coverslips and then labelled with fluorescent BODIPY-Cer. All coverslips were then directly observed by vital fluorescence microscopy. **(A)** Representative images in a healthy donor and a patient. Arrowheads, Cer-enriched domains; arrows, Cer-enriched patches. **(B, C)** Quantification of the proportion of RBCs presenting Cer-enriched patches. Data are expressed as % of healthy RBCs and presented either per patient cohort [means ± SD of 5 or 2 patients; **(B)**] or per individual patient [means ± SD of 3 independent experiments; **(C)**]. Statistical analyses are indicated (i) next to the ChAc bar for the comparison of the whole ChAc cohort vs. the corresponding healthy cohort [**(B)**; one sample t-test; **, p < 0.01]; (ii) next to the concerned bar for individual patients vs. corresponding healthy donors [**(C)**; Kruskal-Wallis test with Dunn’s multiple comparisons test; ns, not significant; *, p < 0.05]; and (iii) above a bracket for the 7 patients together vs*.*the corresponding group of healthy donors [**(C)**; one sample t-test; ^$$$^, p < 0.001]. **(D)** Correlation between the proportion of RBCs with Cer-enriched patches [data from **(C)**] and the abundance of chol-enriched domains [data from Figure 6C]. **(E)** Absence of correlation between the proportion of RBCs with Cer-enriched patches [data from **(C)**] and the abundance of SM-enriched domains [data from Figure 6F].

To determine whether chol- or SM-enriched domain alteration could result from RBC maturation defects, we analyzed the distribution of ceramide, a sphingolipid mainly enriched in domains at healthy RBC membrane (Cloos et al., 2020) but associated in hypobetalipoproteinemia with Cer-enriched patches and related with RBC maturation defects (Cloos et al., 2021). In healthy RBCs, ceramide was mainly found in submicrometric domains, only ∼3% of RBCs showing Cer-enriched patches (Figures 7A–C). In contrast, in ChAc and MLS patients, ∼40% of RBCs showed Cer-enriched patches (Figures 7A, B). The highest increase was seen in ChAc2, 4 and 5 (Figure 7C), *i.e.*, those showing the highest rise of chol-enriched domains. Accordingly, those two parameters very well and positively correlated regardless of including MLS patients in the data or not (Figure 7C vs. Supplementary Figure 2B) but not with the proportion of SM-enriched domains (Figure 7E), supporting our previous hypothesis of differential mechanisms for lipid domain alterations.

## Discussion

We here revealed that RBC morphology, projected surface area, deformability, cytoskeleton, PS transversal asymmetry as well as cholesterol and ceramide lateral distribution were all impaired in neuracanthocytosis syndromes whereas SM lateral distribution in domains was poorly affected. Whereas the origin of the acanthocytes was not elucidated, alterations were all consistent with both RBC maturation defects and membrane impairment. Moreover, lipid distribution analysis well correlated with laboratory parameters typically altered in neuroacanthocytosis and could represent a useful tool to evaluate the disease.

### Limitations of the study

A wide range of research methods was used in this study to assess RBC morphological, cytoskeletal and membrane changes in the two neuroacanthocytosis syndromes as well as their potential consequences for RBC deformability. Nevertheless, our experimental strategy presents some drawbacks. First, for logistic reasons, not all patient samples were available at all investigation sites. For this reason, combined with the fact that imaging experiments are at best semi-quantitative, all the patient data were expressed in % variation or % difference of healthy donors and correlations were made with patient data expressed by reference to the healthy donors.

Second, our study included only 7 patients, among which 5 with ChAc and 2 with MLS. Although this appears acceptable based on the rarity of those 2 diseases (estimated prevalence 1:1,000,000 and 1:10,000,000 respectively) and whereas the two diseases shared common alterations of RBC morphology, projected surface area, deformability, PS transversal asymmetry as well as cholesterol and ceramide lateral distribution, we cannot exclude the possibility of subtle disease-specific changes that were not evidenced in our study. The most evident example relates to the spectrin cytoskeleton, for which the correlation with the RBC projected area was considerably better when taking only the ChAc patients into account, which could suggest differential cytoskeleton alterations in the two syndromes. This possibility remains to be tested on a higher number of patients with MLS syndrome.

Third, on an experimental point-of-view, most of the analyses were performed on RBCs spread on PLL, thus preventing the selection of acanthocytes for a more specific analysis. Moreover, to analyze spectrin, RBCs were opened which could induce some artefacts. Nevertheless, the method was carefully validated and internal controls were used, as described in the Result section. Finally, to analyze lipids, we used fluorescent lipid analogs (for SM and ceramide) or specific toxin fragments (for chol). Here again caution is required since the validity of fluorescent lipid analogs as *bona fide* surrogates of endogenous lipid counterparts has been for a long time debated in view of the bulk fluorophore, which can deeply modify biophysical properties, and toxin fragments are bigger than endogenous lipids, which could induce artificial clustering. Nevertheless, our group carefully validated these two classes of lipid probes in the past (Tyteca et al., 2010; D'Auria et al., 2013; D′auria et al., 2011; Carquin et al., 2014; Carquin et al. 2016; Carquin et al. 2015).

Fourth, we observed a variability in the analyzed parameters between individual patients. This could be attributed to either a too low number of determinations resulting from logistic reasons (e.g., ceramide patches which were highly increased although sometimes non significantly) and/or to a biological difference between patients (e.g., cytoskeleton membrane occupancy, RBC projected area). To circumvent the logistic difficulty, we analyzed all the patients together, revealing significant modifications as compared to healthy donors. We also established correlations between parameters, showing for some parameters excellent coefficients of correlations, particularly in the ChAc cohort.

### Origin of acanthocytes in neuroacanthocytosis syndromes

Several lines of evidence suggest that lipid metabolism and distribution alteration is causal for the development of acanthocytosis. First, hypo- and abeta lipoproteinemia lead to the appearance of acanthocytes (Levy 2015; Cloos et al. 2021). Second, in liver failure where acanthocytosis is observed, irregularities in lipid metabolism, particularly an excess of cholesterol, have been associated with the deformation of RBCs (Sharma, Holman, and Brown 2023). Third, our previous study on ChAc patients revealed lipid changes, including long chain PE and two ceramide species, but also single PC and SM (sub)species (Peikert et al, 2024). Since PE is a non-bilayer forming lipid (de Kruijff, 1997), an increase of longer PE species might at least partially explain the morphological alterations of acanthocytes. Moreover, non-bilayer lipids may affect integration of proteins into membranes, their lateral movement and their function (van den Brink-van der Laan et al., 2004). Fourth, the increase of longer PE in the inner plasma membrane leaflet could in turn impact the clustering of SM and/or cholesterol in domains in the outer leaflet, thereby potentially favoring the formation of acanthocytes. This hypothesis remains to be tested on RBCs labelled in suspension for specific lipids.

### RBC maturation defects as a major feature in neuroacanthocytosis syndromes

As VPS13A is associated with lipid transport from the ER to the plasma membrane, alterations found here might stem from erythroblasts, a erythropoiesis stage at which ER is still present. Indeed, using erythroblast model cells, de Camilli and colleagues have shown that overexpressed VPS13A localizes at ER-plasma membrane contact sites dependently on XK (Amos et al., 2023). Moreover, we observed a ∼2-fold increase in the number of chol-enriched domains in both diseases, despite preservation of the total cholesterol content in ChAc (Peikert et al., 2024). Although we did not prove that this increase resulted from RBC maturation defects, observations on the chol-binding protein stomatin in patients with PKAN, a neurodegenerative condition associated with acanthocytosis and which was formerly grouped under the neuroacanthocytosis umbrella term (Walker and Danek, 2021), support this possibility. Indeed, stomatin, which is associated with chol-enriched membrane regions (Rungaldier et al., 2017) and lost during erythroblast enucleation (Bell et al, 2013), is increased in RBCs from patients with PKAN, as revealed by confocal microscopy (Cluitmans et al., 2015).

Nevertheless, several lines of evidence also support maturation defects during the R1 reticulocyte stage, known to undergo significant rearrangements in reticulocyte membrane and intracellular components in particular via exosome release and mitophagy. First, Lupo et al. have shown by electron microscopy the presence of membrane remnants in circulating ChAc RBCs and the delayed clearance of mitochondria and lysosomes in the reticulocyte-enriched chorea-acanthocytosis red cell fractions (Lupo et al., 2016). Second, the strong increase in the proportion of RBCs showing Cer-enriched patches we found here could correspond to mitochondria remnants, as discussed for hypobetalipoproteinemia in (Cloos et al., 2021) and in agreement with the ceramide enrichment in the outer mitochondrial membrane (Siskind, 2005). Accordingly, lipidomic studies have revealed a slight increase in the content of two ceramide species in ChAc patients, Cer 34:1;2 and Cer 42:2;2 (Peikert et al. 2024). Interestingly, abnormal ceramide levels have been associated with several neurodegenerative conditions (Mielke et al, 2013). Third, longer and more unsaturated PE species are increased in ChAc patients whereas smaller and more saturated PE species are decreased (Peikert et al., 2024), an observation compatible with an RBC maturation defect. Indeed, the relative abundance of longer and more unsaturated PE species decreases during reticulocyte maturation into RBCs whereas smaller and more saturated PE species increase in proportion (Minetti et al., 2025). Moreover, PE is known to play a central role in autophagosome formation and is a regulator of autophagy (Hsu and Shi, 2017), an essential process during erythropoiesis which is impaired in ChAc disease (Lupo et al., 2016; Peikert et al., 2021).

It remains, however, to determine how the loss of function of the bridge-like lipid transfer protein VPS13A and the scramblase XK could affect membrane lipid distribution in RBC maturation defect-related manner. As VPS13A associates with organelle membrane contact sites, its loss of function will in turn affect lipid transfer and related organelle function. Supporting this possibility, it has been shown that ER-mitochondria contact sites are decreased, mitochondria are fragmented and mitophagy is decreased whereas lipid droplet numbers are increased in VPS13A-depleted cells (Yeshaw et al., 2019). In yeast, mutations found in the *VPS13A* gene of ChAc patients have specific defects in the mitochondrial aspect of VPS13 function (Park et al., 2016) and VPS13A is required for efficient lysosomal degradation (Munoz-Braceras et al., 2019). Moreover, the presence of membrane remnants in diseased RBCs suggested that VPS13A plays an important role for membrane removal. Finally, maturation defects might be related to impaired autophagy, as outlined above.

### Impairment of the RBC membrane as an additional feature in some neuroacanthocytosis patients

Whereas RBC maturation defects could explain several lipid alterations described in the present study, our data point to additional impairments of the RBC membrane lipid distribution and morphology, accompanied by a reduction of RBC deformability in agreement with (Reichel et al., 2022) and consistent with the presence of VPS13A at significant levels in mature RBCs (Minetti et al., 2025). Indeed, we showed here that the spectrin cytoskeleton was denser in the patient RBCs. Cytoskeletal alterations and abnormalities were previously revealed through Band3 immunolabelling in neuroacanthocytosis syndrome RBCs (Wong, 2004; Adjobo-Hermans, Cluitmans, and Bosman, 2015) but contrasted with the reduction of the spectrin signals in patients with PKAN revealed by confocal microscopy (Cluitmans et al., 2015). We could reasonably exclude a bias related to our quantification method, as we showed a decrease of the spectrin cytoskeleton occupancy in RBCs from a patient with hypobetalipoproteinemia, another acanthocyte-related disease (Cloos et al., 2021). We instead propose that the increased cytoskeleton membrane occupancy observed here in some patients could be related to their high spheroechinocyte proportion. In agreement with this hypothesis, the two patients without spheroechinocytes, *i.e.*, ChAc2 and ChAc3, had a preserved spectrin network and an almost perfect negative correlation between the projected surface area of RBCs and the spectrin membrane occupancy was found in the ChAc cohort.

Densification of the RBC cytoskeleton in ChAc4 and 5 could in turn explain the decrease of SM-enriched domain abundance found in these patients. Indeed, in healthy RBCs, those domains depend on membrane:cytoskeleton anchorage (Conrard et al., 2018). However, no correlation was found between the abundance of SM-enriched domains and the spectrin cytoskeleton membrane occupancy, suggesting that the spectrin cytoskeleton alteration is probably not the only factor involved in the lateral distribution of SM at the surface of diseased RBCs. The modification of the plasma membrane composition in specific lipid species and PS transversal asymmetry could also contribute to this process. For instance, although no detectable alteration of the SM global content can be revealed in ChAc, subtle changes in SM subspecies have been evidenced, with a tendency to increase for species with long and polyunsaturated fatty acids (≥42 carbons and ≥4 unsaturation) (Peikert et al., 2024). Likewise, in the inner leaflet, the ratio between PE species containing less than 36 carbons and 4 unsaturation and PEs with more than 36 carbons and 4 unsaturation was decreased by ∼2.5-fold in ChAc (Peikert et al., 2024). Finally, PS surface exposure was slightly but significantly decreased in the global patient cohort, as reported in ChAc after RBC stimulation with lysophosphatidic acid (Siegl et al., 2013). This could result from alterations of the RBC membrane which could compensate the expected increased PS contents due to RBC maturation defects (Minetti et al. 2025), resulting into no global changes of PS levels in ChAc (Peikert et al., 2024).

### Correlation of lipid distribution with laboratory parameters

**FIGURE 8:**
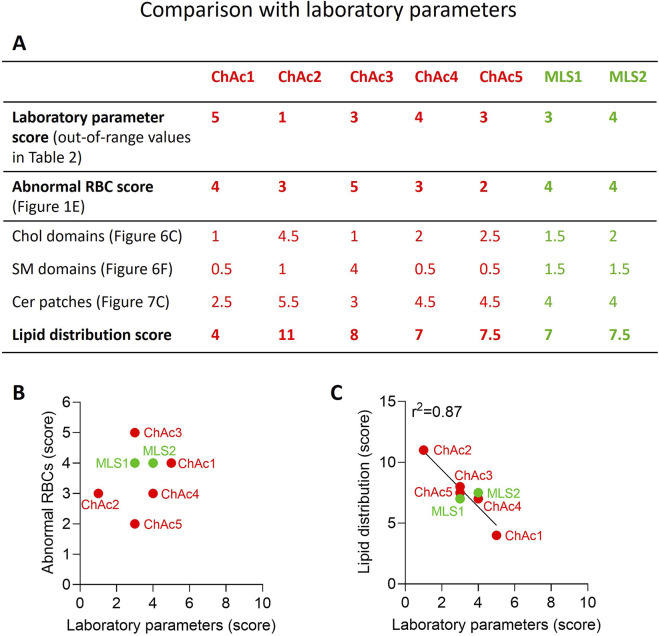
Lipid distribution score correlates with laboratory parameters in contrast to abnormal RBC count. **(A)** Scores for alterations of laboratory parameters, abnormal RBC proportion and lipid distribution. The procedure used to determine those 3 scores is described in details in the Material and Method section. The higher the score, the higher the laboratory parameters alteration, the higher the abnormal RBC proportion and the higher the increase of lipid domains/patches as compared to healthy RBCs. **(B)** Absence of correlation between laboratory parameters and abnormal RBCs scores. **(C)** Correlation between laboratory parameters and lipid distribution scores.

Whereas ChAc and MLS cohorts cannot be distinguished based on laboratory parameters (Figure 8A), there were differences between patients: ChAc2 was the less affected, ChAc1 the most affected and the only one to have an increase of reticulocytes, and ChAc3 the only one to present an elevated MCHC. Surprisingly, the most affected patient ChAc1 was the only one with a weak chorein (VPS13A) band whereas the other patients showed no chorein band in Western blot. Moreover, no relation between laboratory parameters and alterations of RBC morphology could be evidenced (Figures 8A, B). We, therefore, asked if and how evaluation of lipid distribution could be more powerful for better understanding and evaluating neuroacanthocytosis. The answer was no for transversal asymmetry evaluated by Annexin V, as it was even lower than in healthy RBCs. In contrast, the abundance of chol-enriched domains and the proportion of RBCs with Cer-enriched patches could be useful for the following reasons. First, both parameters were significantly increased in the global disease cohort as compared to healthy donors. Second, there were differences between patients for the two parameters and these varied in the same way but at different levels. Third, the presence of Cer-enriched patches suggested RBC maturation defect, as discussed above. Fourth, the correlation between these two parameters based on the seven patients could be extended to one patient with hypobetalipoproteinemia (Supplementary Figure 3). Based on the differential modulation of SM-enriched domains in the different patients and the fact that this modification could reflect an alteration of the RBC membrane itself rather than a maturation defect, we also included this lipid parameter in the score calculation. The lowest score was obtained for ChAc1, the most affected patient based on laboratory parameters and RBC morphology. Conversely, the highest score was obtained for ChAc2, the least affected patient clinically and showing limited alteration of RBC morphology (Figure 8A). This suggested that the greater the abundance of RBC surface domains and Cer-enriched patches, the lower the laboratory parameters alterations and the fragility of the RBC membrane. Remarkably, we obtained an excellent inverse correlation with the laboratory parameters score regardless of including both ChAc and MLS patients or only ChAc patients in the data (Figure 8C vs. Supplementary Figure 2C). This suggested that lipid distribution could represent a more reliable tool than abnormal RBC measurement for neuroacanthocytosis, e.g., for disease monitoring or as an outcome biomarker for clinical studies.

### Conclusion and overall significance

We revealed the impairment of lateral distribution of specific lipids in RBCs from both ChAc and MLS cohorts. Both RBC maturation defects and impairments of the RBC membrane appeared to contribute to the pathophysiology of the diseases. As all shown phenotypes here were quite similar/overlapping in VPS13A and XK diseases, one could argue that mainly the absence of this complex contributed to the RBC phenotype. Of note, the presence of VPS13A in XK disease and the presence of XK in VPS13A disease, respectively, cannot compensate/rescue the phenotype. We also showed that the extent of lipid distribution alteration well correlated with laboratory parameters, opening the way to consider lipid status in the evaluation of the disease.

## Data Availability

The original contributions presented in the study are included in the article/Supplementary Material, further inquiries can be directed to the corresponding author.

## References

[B1] Adjobo-Hermans M. J. Cluitmans J. C. Bosman G. J. (2015). Neuroacanthocytosis: observations, theories and perspectives on the origin and significance of acanthocytes. Tremor Other Hyperkinet Mov. (N Y) 5, 328. 10.7916/D8VH5N2M 26317043 PMC4548760

[B2] Amos C. Xu P. De Camilli P. (2023). 'Erythroid differentiation dependent interaction of VPS13A with XK at the plasma membrane of K562 cells. Contact (Thousand Oaks) 6, 25152564231215133. 10.1177/25152564231215133 38144430 PMC10748539

[B5555] Bell A. J. Satchwell T. J. Heesom K. J. Achilli C. Hawley B. R. Kupzig S. Hazell M.(2013). Protein distribution during human erythroblast enucleation in vitro. PLoS ONE 8 (4), e60300. 10.1371/journal.pone.0060300 23565219 PMC3614867

[B3] Baines A. J. (2010). The spectrin-ankyrin-4.1-adducin membrane skeleton: adapting eukaryotic cells to the demands of animal life. Protoplasma 244, 99–131. 10.1007/s00709-010-0181-1 20668894

[B4] Braschi B. Bruford E. A. Cavanagh A. T. Neuman S. D. Bashirullah A. (2022). The bridge-like lipid transfer protein (BLTP) gene group: introducing new nomenclature based on structural homology indicating shared function. Hum. Genomics 16, 66. 10.1186/s40246-022-00439-3 36461115 PMC9719229

[B5] Carquin M. Conrard L. Pollet H. Van Der Smissen P. Cominelli A. Veiga-da-Cunha M. (2015). 'Cholesterol segregates into submicrometric domains at the living erythrocyte membrane: evidence and regulation. Cell. Mol. Life Sci. 72, 4633–4651. 10.1007/s00018-015-1951-x 26077601 PMC11113096

[B6] Carquin M. D'Auria L. Pollet H. Bongarzone E. R. Tyteca D. (2016). Recent progress on lipid lateral heterogeneity in plasma membranes: from rafts to submicrometric domains. Prog. Lipid Res. 62, 1–24. 10.1016/j.plipres.2015.12.004 26738447 PMC4851880

[B7] Carquin M. Pollet H. Veiga-da-Cunha M. Cominelli A. Van Der Smissen P. N'Kuli F. (2014). 'Endogenous sphingomyelin segregates into submicrometric domains in the living erythrocyte membrane. J. Lipid Res. 55, 1331–1342. 10.1194/jlr.M048538 24826836 PMC4076090

[B9] Cloos A. S. Daenen L. G. M. Maja M. Stommen A. Vanderroost J. Van Der Smissen P. (2021). 'Impaired cytoskeletal and membrane biophysical properties of acanthocytes in hypobetalipoproteinemia - a case study. Front. Physiol. 12, 638027. 10.3389/fphys.2021.638027 33708142 PMC7940373

[B10] Cloos A. S. Ghodsi M. Stommen A. Vanderroost J. Dauguet N. Pollet H. (2020). 'Interplay between plasma membrane lipid alteration, oxidative stress and calcium-based mechanism for extracellular vesicle biogenesis from erythrocytes during blood storage. Front. Physiol. 11, 712. 10.3389/fphys.2020.00712 32719614 PMC7350142

[B11] Cloos A.-S. Van Der Smissen P. Mignolet E. Larondelle Y. Terrasi R. Muccioli G. G. (2023). Red blood cells from patients with sitosterolemia exhibit impaired membrane lipid composition and distribution and decreased deformability. Front. Hematol. 2, 055086. 10.3389/frhem.2023.1055086

[B12] Cluitmans J. C. Tomelleri C. Yapici Z. Dinkla S. Bovee-Geurts P. Chokkalingam V. (2015). Abnormal red cell structure and function in neuroacanthocytosis. PLOS One 10, e0125580. 10.1371/journal.pone.0125580 25933379 PMC4416783

[B13] Conrard L. Stommen A. Cloos A. S. Steinkuhler J. Dimova R. Pollet H. (2018). Spatial relationship and functional relevance of three lipid domain populations at the erythrocyte surface. Cell Physiol. Biochem. 51, 1544–1565. 10.1159/000495645 30497064

[B14] Conrard L. Tyteca D. (2019). Regulation of membrane calcium transport proteins by the surrounding lipid environment. Biomolecules 9, 513. 10.3390/biom9100513 31547139 PMC6843150

[B15] Darras A. Peikert K. Rabe A. Yaya F. Simionato G. John T. (2021). Acanthocyte sedimentation rate as a diagnostic biomarker for neuroacanthocytosis syndromes: experimental evidence and physical justification. Cells 10, 788. 10.3390/cells10040788 33918219 PMC8067274

[B16] D'Auria L. Fenaux M. Aleksandrowicz P. Van Der Smissen P. Chantrain C. Vermylen C. (2013). Micrometric segregation of fluorescent membrane lipids: relevance for endogenous lipids and biogenesis in erythrocytes. J. lipid Res. 54, 1066–1076. 10.1194/jlr.M034314 23322884 PMC3605983

[B17] D′auria L. Van Der Smissen P. Bruyneel F. Courtoy P. J. Tyteca D. (2011). Segregation of fluorescent membrane lipids into distinct micrometric domains: evidence for phase compartmentation of natural lipids? PLOS One 6, e17021–e21. 10.1371/journal.pone.0017021 21386970 PMC3046177

[B18] De Franceschi L. Tomelleri C. Matte A. Brunati A. M. Bovee-Geurts P. H. Bertoldi M. (2011). 'Erythrocyte membrane changes of chorea-acanthocytosis are the result of altered Lyn kinase activity. Blood 118, 5652–5663. 10.1182/blood-2011-05-355339 21951684 PMC3217364

[B19] de Kruijff B. (1997). Lipid polymorphism and biomembrane function. Curr. Opin. Chem. Biol. 1, 564–569. 10.1016/s1367-5931(97)80053-1 9667894

[B20] Dobson-Stone C. Velayos-Baeza A. Filippone L. A. Westbury S. Storch A. Erdmann T. (2004). 'Chorein detection for the diagnosis of chorea-acanthocytosis. Ann. Neurol. 56, 299–302. 10.1002/ana.20200 15293285

[B21] Foller M. Hermann A. Gu S. Alesutan I. Qadri S. M. Borst O. (2012). Chorein-sensitive polymerization of cortical actin and suicidal cell death in chorea-acanthocytosis. Faseb J. 26, 1526–1534. 10.1096/fj.11-198317 22227296

[B22] Ghodsi M. Cloos A.-S. Mozaheb N. Van Der Smissen P. Henriet P. Pierreux C. E. (2023). “Variability of extracellular vesicle release during storage of red blood cell concentrates is associated with differential membrane alterations, including loss of cholesterol-enriched domains.”, Front. Physiol. 14, 1–20. 10.3389/fphys.2023.1205493 PMC10318158 37408586

[B23] Guillén-Samander A. Wu Y. Pineda S. S. García F. J. Eisen J. N. Leonzino M. (2022). A partnership between the lipid scramblase XK and the lipid transfer protein VPS13A at the plasma membrane. Proc. Natl. Acad. Sci. U. S. A. 119, e2205425119. 10.1073/pnas.2205425119 35994651 PMC9436381

[B24] Hanna M. Guillen-Samander A. De Camilli P. (2023). RBG motif bridge-like lipid transport proteins: structure, functions, and open questions. Annu. Rev. Cell Dev. Biol. 39, 409–434. 10.1146/annurev-cellbio-120420-014634 37406299

[B25] Hsu P. Shi Y. (2017). Regulation of autophagy by mitochondrial phospholipids in health and diseases. Biochim. Biophys. Acta Mol. Cell Biol. Lipids 1862, 114–129. 10.1016/j.bbalip.2016.08.003 27502688 PMC7707390

[B26] Jung H. H. Danek A. Walker R. H. Frey B. M. Peikert K. (1993). “'McLeod neuroacanthocytosis syndrome,” in: GeneReviews® [Internet] Editors Adam M. P. Feldman J. Mirzaa G. M. Pagon R. A. Wallace S. E. Bean L. J. H. Seattle (WA): University of Washington, Seattle. 20301528

[B27] Kapus A. Janmey P. (2013). Plasma membrane--cortical cytoskeleton interactions: a cell biology approach with biophysical considerations. Compr. Physiol. 3, 1231–1281. 10.1002/cphy.c120015 23897686

[B28] Kihm A. Kaestner L. Wagner C. Quint S. (2018). Classification of red blood cell shapes in flow using outlier tolerant machine learning. PLoS Comput. Biol. 14, e1006278. 10.1371/journal.pcbi.1006278 29906283 PMC6021115

[B29] Kumar N. Leonzino M. Hancock-Cerutti W. Horenkamp F. A. Li P. Lees J. A. (2018). VPS13A and VPS13C are lipid transport proteins differentially localized at ER contact sites. J. Cell Biol. 217, 3625–3639. 10.1083/jcb.201807019 30093493 PMC6168267

[B30] Leonard C. Alsteens D. Dumitru A. C. Mingeot-Leclercq M. P. Tyteca D. (2017a). “Lipid domains and membrane (re)shaping: from biophysics to biology,” in The role of the physical properties of membranes in influencing biological phenomena. Editors Ruysschaert J. M. Epand R. (Springer). series in biophysics).

[B31] Leonard C. Conrard L. Guthmann M. Pollet H. Carquin M. Vermylen C. (2017b). 'Contribution of plasma membrane lipid domains to red blood cell (re)shaping. Sci. Rep. 7, 4264. 10.1038/s41598-017-04388-z 28655935 PMC5487352

[B32] Leonard C. Pollet H. Vermylen C. Gov N. Tyteca D. Mingeot-Leclercq M. P. (2018). Tuning of differential lipid order between submicrometric domains and surrounding membrane upon erythrocyte reshaping. Cell Physiol. Biochem. 48, 2563–2582. 10.1159/000492700 30121671

[B33] Levy E. (2015). Insights from human congenital disorders of intestinal lipid metabolism. J. Lipid Res. 56, 945–962. 10.1194/jlr.R052415 25387865 PMC4409285

[B34] Lupo F. Tibaldi E. Matte A. Sharma A. K. Brunati A. M. Alper S. L. (2016). A new molecular link between defective autophagy and erythroid abnormalities in chorea-acanthocytosis. Blood 128, 2976–2987. 10.1182/blood-2016-07-727321 27742708 PMC5179337

[B67] Mielke M. M. Maetzler W. Haughey N. J. Bandaru V. V. Savica R. Deuschle C.(2023). Plasma ceramide and glucosylceramide metabolism is altered in sporadic Parkinson's disease and associated with cognitive impairment: a pilot study PLoS One. 8, 9–1541. 10.1371/journal.pone.0073094 PMC3776817 24058461

[B35] Miltenberger-Miltenyi G. Jones A. Tetlow A. M. Conceição V. A. Crary J. F. Ditzel R. M. (2023). Sphingolipid and phospholipid levels are altered in human brain in chorea-acanthocytosis. Mov. Disord. 38, 1535–1541. 10.1002/mds.29445 37307400

[B68] Minetti G. Dorn I. Köfeler H. Perotti C. Kaestner L. (2025). Insights from lipidomics into the terminal maturation of circulating human reticulocytes. Cell Death Discov. 11, 79. 10.1038/s41420-025-02318-x 40016214 PMC11868425

[B39] Munoz-Braceras S. Tornero-Ecija A. R. Vincent O. Escalante R. (2019). VPS13A is closely associated with mitochondria and is required for efficient lysosomal degradation. Dis. Model Mech. 12, dmm036681. 10.1242/dmm.036681 30709847 PMC6398486

[B40] Park J. S. Neiman A. M. (2020). XK is a partner for VPS13A: a molecular link between Chorea-Acanthocytosis and McLeod Syndrome. Mol. Biol. Cell 31, 2425–2436. 10.1091/mbc.E19-08-0439-T 32845802 PMC7851852

[B41] Park J. S. Thorsness M. K. Policastro R. McGoldrick L. L. Hollingsworth N. M. Thorsness P. E. (2016). 'Yeast Vps13 promotes mitochondrial function and is localized at membrane contact sites. Mol. Biol. Cell 27, 2435–2449. 10.1091/mbc.E16-02-0112 27280386 PMC4966984

[B42] Peikert K. Dobson-Stone C. Rampoldi L. Miltenberger-Miltenyi G. Neiman A. De Camilli P. (1993). “'VPS13A disease,” Editors Adam M. P. Feldman J. Mirzaa G. M. Pagon R. A. Wallace S. E. Bean L. J. H. GeneReviews. 20301561

[B43] Peikert K. Dobson-Stone C. Rampoldi L. Miltenberger-Miltenyi G. Neiman A. De Camilli P. (2002). “' VPS13A disease,”1993. Seattle (WA)): University of Washington, Seattle. 20301561

[B44] Peikert K. Federti E. Matte A. Constantin G. Pietronigro E. C. Fabene P. F. (2021). Therapeutic targeting of Lyn kinase to treat chorea-acanthocytosis. Acta Neuropathol. Commun. 9, 81. 10.1186/s40478-021-01181-y 33941276 PMC8091687

[B45] Peikert K. Hermann A. Danek A. (2022). XK-associated McLeod syndrome: nonhematological manifestations and relation to VPS13A disease. Transfus. Med. Hemother 49, 4–12. 10.1159/000521417 35221863 PMC8832239

[B46] Peikert K. Spranger A. Miltenberger-Miltenyi G. Glass H. Falkenburger B. Klose C. (2024). Phosphatidylethanolamines are the main lipid class altered in red blood cells from patients with VPS13A disease/chorea-acanthocytosis. Mov. Disord. 10.1002/mds.30086 PMC11926492 39665525

[B47] Phillips G. R. Saville J. T. Hancock S. E. Brown S. H. J. Jenner A. M. McLean C. (2022). The long and the short of Huntington's disease: how the sphingolipid profile is shifted in the caudate of advanced clinical cases. Brain Commun. 4, fcab303. 10.1093/braincomms/fcab303 35169703 PMC8833324

[B48] Pollet H. Cloos A. S. Stommen A. Vanderroost J. Conrard L. Paquot A. (2020). Aberrant membrane composition and biophysical properties impair erythrocyte morphology and functionality in elliptocytosis. Biomolecules 10, 1120. 10.3390/biom10081120 32751168 PMC7465299

[B49] Pollet H. Conrard L. Cloos A. S. Tyteca D. (2018). Plasma membrane lipid domains as platforms for vesicle biogenesis and shedding? Biomolecules 8, 94. 10.3390/biom8030094 30223513 PMC6164003

[B50] Rabe A. Kihm A. Darras A. Peikert K. Simionato G. Dasanna A. K. (2021). Prototype foamy virus integrase displays unique biochemical activities among retroviral integrases. Biomolecules 11, 1910. 10.3390/biom11121910 34944553 PMC8699820

[B51] Recktenwald S. M. Lopes M. G. M. Peter S. Hof S. Simionato G. Peikert K. (2022). 'Erysense, a lab-on-a-chip-based point-of-care device to evaluate red blood cell flow properties with multiple clinical applications. Front. Physiol. 13, 884690. 10.3389/fphys.2022.884690 35574449 PMC9091344

[B52] Reichel F. Krater M. Peikert K. Glass H. Rosendahl P. Herbig M. (2022). Changes in blood cell deformability in chorea-acanthocytosis and effects of treatment with dasatinib or lithium. Front. Physiol. 13, 852946. 10.3389/fphys.2022.852946 35444561 PMC9013823

[B53] Rungaldier S. Umlauf E. Mairhofer M. Salzer U. Thiele C. Prohaska R. (2017). Structure-function analysis of human stomatin: a mutation study. PLOS One 12, e0178646. 10.1371/journal.pone.0178646 28575093 PMC5456319

[B54] Ryoden Y. Segawa K. Nagata S. (2022). “Requirement of Xk and Vps13a for the P2X7-mediated phospholipid scrambling and cell lysis in mouse T cells”, Proc. Natl. Acad. Sci. U. S. A, e2119286119. 10.1073/pnas.2119286119 PMC8851519 35140185

[B55] Salomao M. Zhang X. Yang Y. Lee S. Hartwig J. H. Chasis J. A. (2008). 'Protein 4.1R-dependent multiprotein complex: new insights into the structural organization of the red blood cell membrane. Proc. Natl. Acad. Sci. U. S. A. 105, 8026–8031. 10.1073/pnas.0803225105 18524950 PMC2430353

[B56] Sharma R. Holman C. J. Brown K. E. (2023). 'A thorny matter: spur cell anemia. Ann. Hepatol. 28, 100771. 10.1016/j.aohep.2022.100771 36241039

[B57] Siegl C. Hamminger P. Jank H. Ahting U. Bader B. Danek A. (2013). 'Alterations of red cell membrane properties in neuroacanthocytosis. PLoS One 8, e76715. 10.1371/journal.pone.0076715 24098554 PMC3789665

[B58] Siskind L. J. (2005). Mitochondrial ceramide and the induction of apoptosis. J. Bioenerg. Biomembr. 37, 143–153. 10.1007/s10863-005-6567-7 16167171 PMC2246044

[B60] Stommen A. Ghodsi M. Cloos A. S. Conrard L. Dumitru A. C. Henriet P. (2023). Piezo1 regulation involves lipid domains and the cytoskeleton and is favored by the stomatocyte-discocyte-echinocyte transformation. Biomolecules 14, 51. 10.3390/biom14010051 38254651 PMC10813235

[B61] Tyteca D. D'Auria L. Van Der Smissen P. Medts T. Carpentier S. Monbaliu J. C. (2010). Three unrelated sphingomyelin analogs spontaneously cluster into plasma membrane micrometric domains. Biochimica Biophysica Acta (BBA) - Biomembr. 1798, 909–927. 10.1016/j.bbamem.2010.01.021 20123084

[B62] van den Brink-van der Laan E. Killian J. A. de Kruijff B. (2004). Nonbilayer lipids affect peripheral and integral membrane proteins via changes in the lateral pressure profile. Biochim. Biophys. Acta 1666, 275–288. 10.1016/j.bbamem.2004.06.010 15519321

[B63] Walker R. H. Danek A. (2021). Neuroacanthocytosis - overdue for a taxonomic update. Tremor Other Hyperkinet Mov. (N Y) 11, 1. 10.5334/tohm.583 33510935 PMC7805383

[B64] Walker R. H. Peikert K. Jung H. H. Hermann A. Danek A. (2023). Neuroacanthocytosis syndromes: the clinical perspective. Contact (Thousand Oaks) 6, 25152564231210339. 10.1177/25152564231210339 38090146 PMC10714877

[B65] Wong P. (2004). A basis of the acanthocytosis in inherited and acquired disorders. Med. Hypotheses 62, 966–969. 10.1016/j.mehy.2003.12.032 15142658

[B66] Yeshaw W. M. van der Zwaag M. Pinto F. Lahaye L. L. Faber A. I. Gomez-Sanchez R. (2019). Human VPS13A is associated with multiple organelles and influences mitochondrial morphology and lipid droplet motility. Elife 8, e43561. 10.7554/eLife.43561 30741634 PMC6389287

