# Supplementary figures and images for: Red blood cell lipid distribution in the pathophysiology and laboratory evaluation of chorea-acanthocytosis and McLeod syndrome patients
Source: Front Physiol. 2025 Mar 27;16:1543812. doi: 10.3389/fphys.2025.1543812 (PMC11983514; doi:10.3389/fphys.2025.1543812)

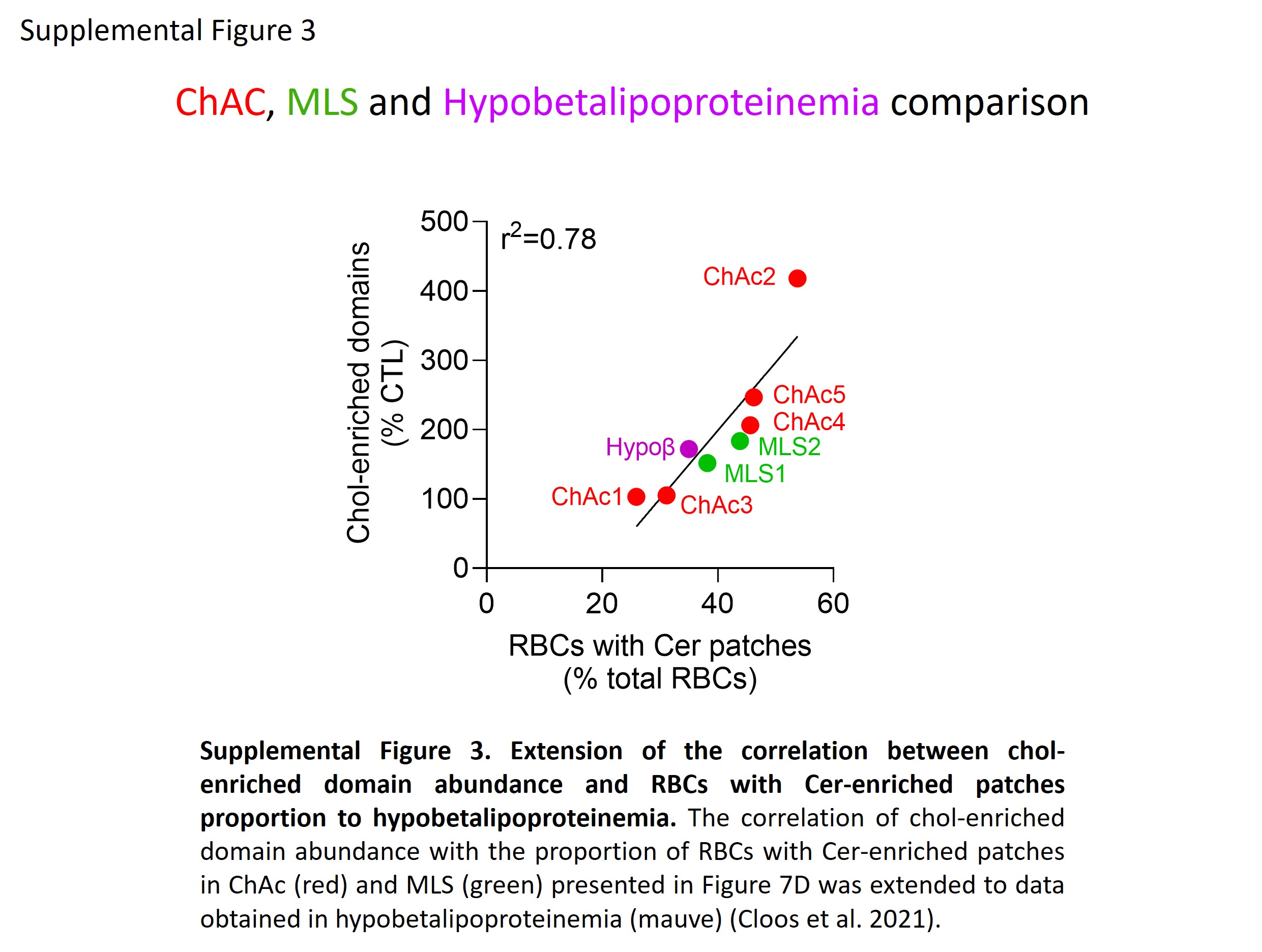

Supplement: Supplementary file 1 [file Image3.jpeg]

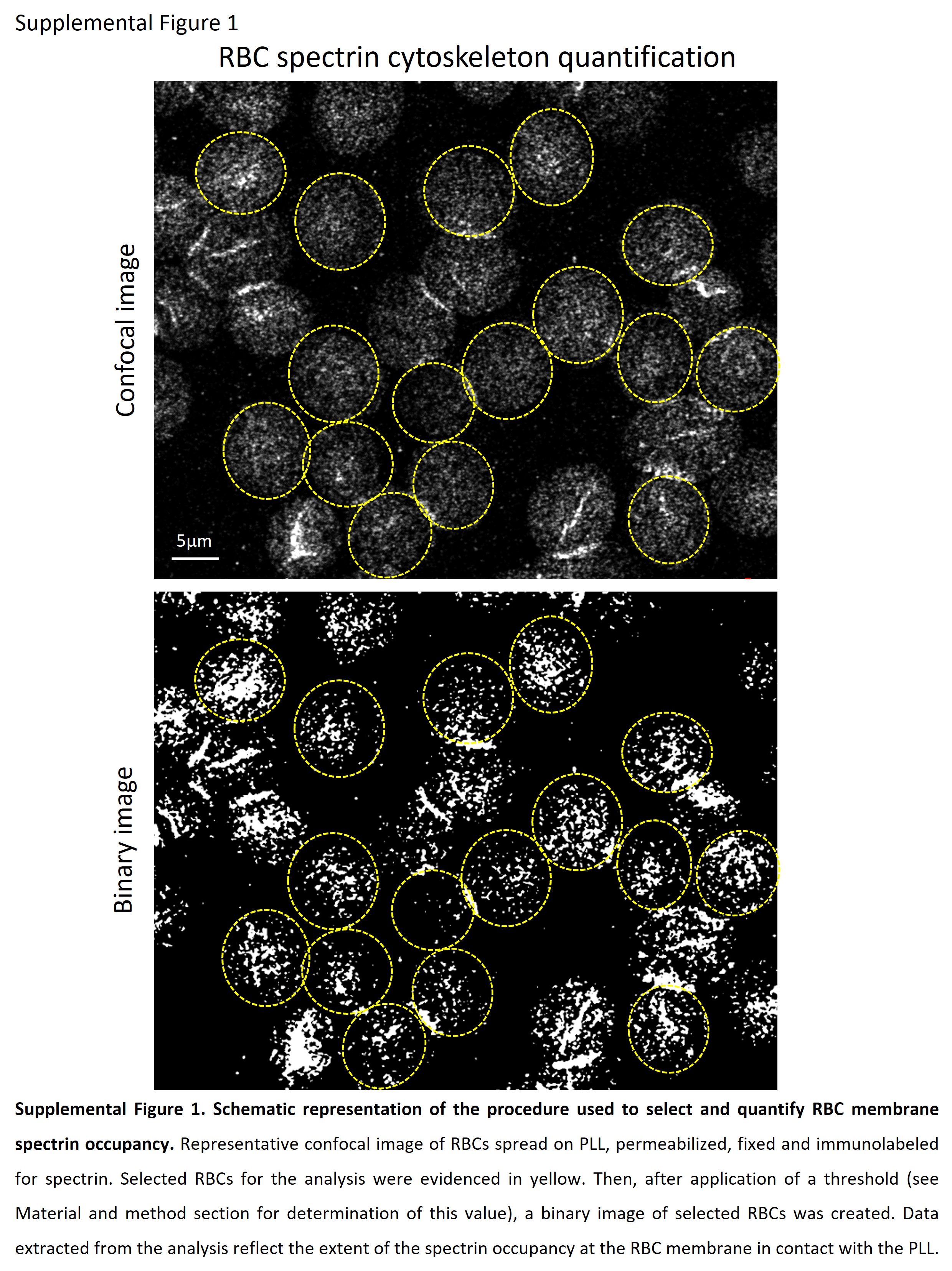

Supplement: Supplementary file 2 [file Image1.jpeg]

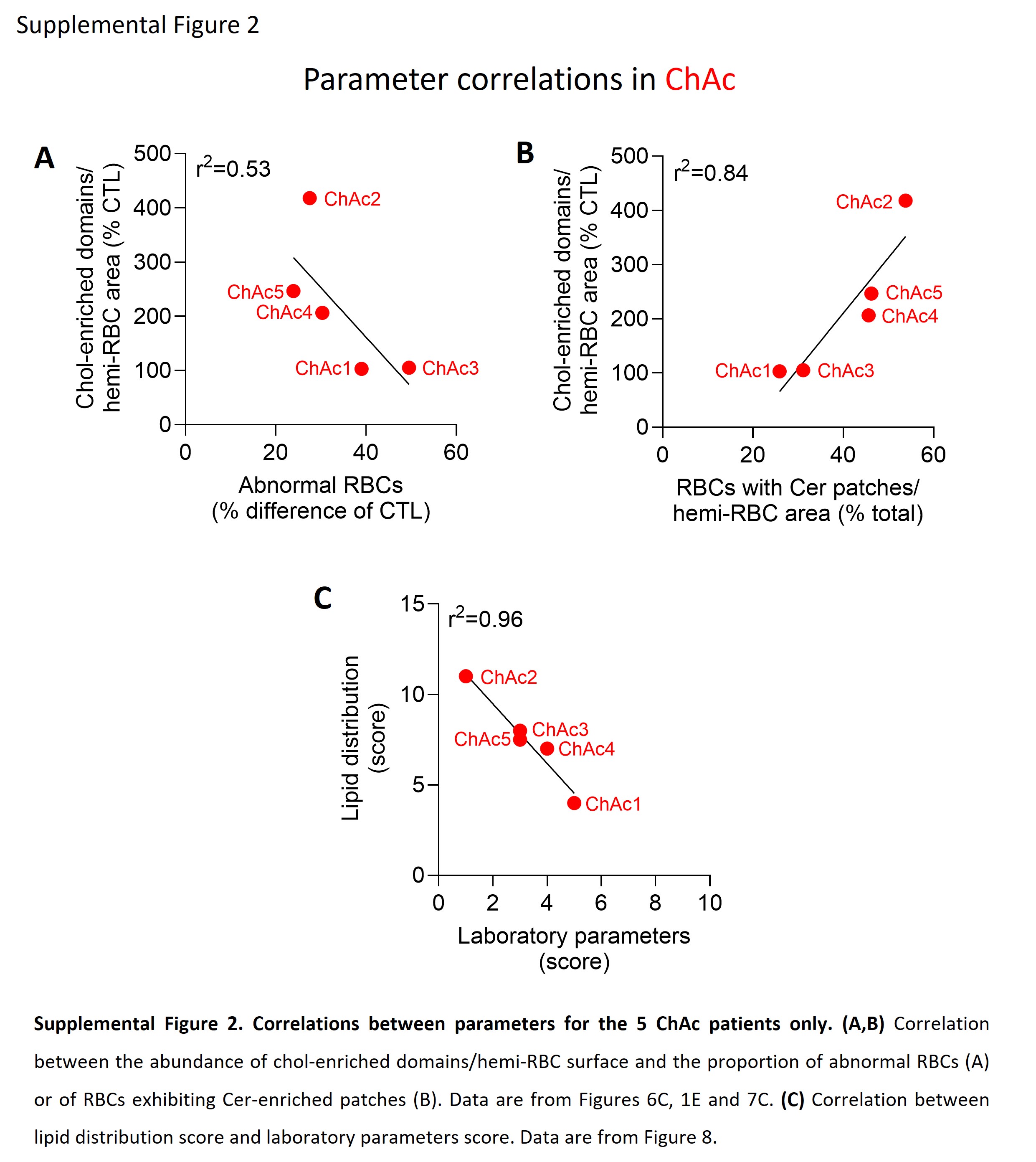

Supplement: Supplementary file 3 [file Image2.jpeg]
